# Improvement of the antioxidant activity, phytochemicals, and cannabinoid compounds of *Cannabis sativa* by salicylic acid elicitor

**DOI:** 10.1002/fsn3.2643

**Published:** 2021-10-25

**Authors:** Elham Mirzamohammad, Abolfazl Alirezalu, Kazem Alirezalu, Asadaolah Norozi, Afsaneh Ansari

**Affiliations:** ^1^ Department of Biology Faculty of Sciences Urmia University Urmia Iran; ^2^ Department of Horticultural Sciences Faculty of Agriculture Urmia University Urmia Iran; ^3^ Department of Food Science and Technology Ahar Faculty of Agriculture and Natural Resources University of Tabriz Tabriz Iran

**Keywords:** cannabinoids, GC‐Mass, phenolic compounds, photosynthetic pigments, salicylic acid

## Abstract

Recently, due to the valuable and high level of phytochemical compounds such as cannabinoids and other secondary metabolites, the cultivation of *Cannabis sativa* has increased in the world. The current study was conducted to evaluate the potential role of exogenous salicylic acid (control, 0.01, 0.1, and 1 M) on enhanced production of pharmaceutically important phytochemicals. The sprayed aerial parts were evaluated based on phenolic (TPC) and flavonoids (TFC) contents, antioxidant capacity (by FRAP and DPPH assay), photosynthetic pigments including chlorophyll a, b (Chl a and Chl b), total carotenoids (TCC), and cannabinoid compounds. Quantification of aerial parts metabolites was performed using gas chromatography. The results indicated that phytochemical compounds and antioxidant capacity in *C. sativa* were influenced by various concentrations of salicylic acid (SA). The highest TPC, TFC, TCC, Chl a, Chl b, and antioxidant capacity were obtained in 1 M treatment, whereas the lowest of them were found in control plants. The major cannabinoids in the analyzed extracts were CBD (19.91%–37.81%), followed by Δ^9^‐THC (10.04%–22.84%), and CBL (nd‐14.78%). The highest CBD (37.81%) and Δ^9^‐THC (22.84%) were obtained in 1 M of SA. These results suggest that the elicitor SA (especially 1 M) was able to improve antioxidant capacity, phytochemicals, and cannabinoid compounds.

## Graphical abstract

Recently, due to the valuable and high level of phytochemical compounds such as cannabinoids and other secondary metabolites, the cultivation of Cannabis sativa has increased in the world. Results of present study suggest that the elicitor SA (especially 1 M) was able to improve antioxidant activity, phytochemicals, and cannabinoid compounds of Cannabis sativa.

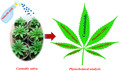

## 1 INTRODUCTION

*Cannabis sativa* L., commonly known as marijuana, is an annual dioecious herb from eastern Asia belonging to the family Cannabaceae (Russo, 2007). *Cannabis* is a remarkable medicinal herb containing many valuable natural components such as cannabinoids, terpenes, phenolics, carotenoids, alkaloids, and amino acids (Andre et al., 2016; Elsohly, 2007). *Cannabis* has been used for medicinal purposes, for treating pain, spasms, asthma, insomnia, depression, and loss of appetite, in many cultures for hundreds of years (Fattore, 2015).

Cannabinoids are the most important chemical compounds found in the *C. sativa* species, including Δ^9^‐tetrahydrocannabinol (Δ^9^‐THC), cannabidiol (CBD), cannabinol (CBN), cannabigerol (CBG), and cannabichromene (CBC) (Elsohly, 2007). The amount and different types of produced cannabinoid compounds determine the class of *Cannabis* species (medicinal‐type or fiber‐type). Several previous studies have reported different types of cannabinoids *C. sativa* plant (De Petrocellis et al., 2011; Elsohly, 2007; Jalali et al., 2019). The two most common and most pharmaceutically relevant compounds of the *C. sativa* plant are CBD and Δ^9^‐THC, which is an isomer of THC. Over the past two decades, a physiologically important drug target for the emergence of the endocannabinoid system has been associated with the effects of *Cannabis* (Mechoulam et al., 2014). Δ9‐THC is the main cause of *C*. *sativa* psychedelic effects, while CBD is a nonpsychoactive compound. Nevertheless, CBD, which is medically used in combination with Δ9‐THC in cannabis‐based drugs, contains a balanced content of both to manage the neuropathic symptoms related to multiple sclerosis (Fernandez, 2016). CBD is the single drug recently generating notable interest due to its useful antiepileptic (Wright et al., 2015), neuroprotective (Ibeas Bih et al., 2015), hypoxia–ischemia (Mori et al., 2017), anxiolytic (Schier et al., 2014), analgesic (Maione et al., 2011), anti‐inflammatory (Burstein, 2015), anti‐asthmatics (Vuolo et al., 2015), antipsychotic (Bhattacharyya et al., 2010), and antitumor activity (Massi et al., 2013) among others.

The salicylic acid (SA) is classified as a phenolic growth regulator or natural plant phenolics having key role in regulation plant growth and development functions (Szepesi et al., 2008). Salicylic acid is a potent signaling molecule that participates in the plant defense mechanism by regulating a broad spectrum of phytochemical and physiological processes (Gunes et al., 2007; Nazar et al., 2011). Estaji and Niknam (2020) revealed that the foliar application of salicylic acid can be considered as a practical strategy for improving secondary metabolites in *Silybum marianum* L. plant such as carbohydrates, antioxidant activity, sucrose, and phenolic compounds. Brito et al. (2019) suggested that the applying proper concentrations of salicylic acid is a useful tool to regulate the cellular homeostasis and growth of plant species. Ghasemi Pirbalouti et al. (2019) proved that the exogenous SA spray applications can remarkably improve some secondary metabolites especially terpenes in *Melissa officinalis* species. Khalili et al. (2009) stated that the accumulation of phytochemicals and antioxidant activity might be induced by exogenous SA application, in *Silybum marianum*.

Recently, due to the high level of phytochemical compounds such as cannabinoids and other secondary metabolites, the cultivation of *Cannabis sativa* has increased in the world. These phytochemicals are substantial and valuable for the pharma industry. The present study was designed to evaluate the potential effects of SA on enhanced production of pharmaceutically important phytochemicals. The treated aerial parts were evaluated based on phenolic and flavonoids contents, antioxidant capacity (by DPPH and FRAP assays), photosynthetic pigments, and cannabinoid compounds. Quantification of aerial parts metabolites was performed using gas chromatography.

## 2 MATERIAL AND METHODS

### 2.1 Plant material

This research was conducted in research greenhouses and laboratories of the Department of Horticultural Sciences at Urmia University from 2017 to 2018. *Cannabis sativa* seeds were first planted in seedling production trays in the greenhouse of Horticultural Sciences, and after the required care and supervision, 90% of the seeds germinated in ten days. After that, the seedlings were transferred 1 month later to the original 16‐size pots for the main experiment. During the experiment, a regular feeding of 20, 20, and 20 ml as much as 200 ml was applied for each pot. The used growing media was a combination of peat moss and perlite with a ratio of 70 to 30.

### 2.2 The studied treatments and the experimental design specifications

The studied treatments included 4 different concentrations of salicylic acid, including zero (control), 0.01, 0.1, and 1 molar in three replications. The treatments were performed by spraying in two stages, the first stage at 10 to 12 leaves and the second stage at the beginning of bud flowering.

### 2.3 Extraction of plant samples

The aerial parts were reaped during flowering and powdered with liquid nitrogen, and then, methanol extraction was carried out using ultrasonic device. One g of each sample was placed in 50 ml falcons, and after adding 20 ml of 80% methanol, the extraction was conducted for half an hour at ultrasonic 30℃ and 120 Hz (Elmasonic) (Alirezalu et al., 2018).

### 2.4 The measurement of total phenolic content (TPC)

The total phenolics of the extracts were measured using a Folin–Ciocalteu reagent. 25 μL of extract were taken from the main extracted solution, and then 1.6 ml of deionized water was added to it. In the next step, 1.2 ml of folin were put into the combination, and after 5 min, 2 ml of 7.5% sodium carbonate was added to it. The samples were then placed at room temperature for 45–45 min. Finally, the absorption at a wavelength of 760 nm was read by a spectrophotometer (MODEl: UV2100 PC). The standard curve based on gallic acid, and drawing and results as mg GAE/g dw (mg/milligrams of gallic acid per g of dry weight) were reported (Singleton et al., 1999).

### 2.5 The measurement of total flavonoid (TFC)

To measure the total flavonoid content of 100 μl of the considered methanol extract, 150 μL of 5% sodium nitrite was added, and after 5 min, 300 μl of 10% aluminum chloride was added. Then, after 5 min, 1 ml of 1 molar solution was added to each sample and distilled water was reached to 5 ml. The absorption wavelength of the solution was read at 380 nm and compared with the control solution. Quercetin solution was used to draw the standard curve, and the total flavonoid content of the extract was reported according to mg Qu/g dw (mg quercetin per g of dry weight) (Chantiratikul et al., 2009).

### 2.6 The measurement of antioxidant capacity by DPPH method

To measure the antioxidant capacity by DPPH, 25 μl of the original methanol extract were poured into a test tube and 2000 μl of DPPH solution were added. The resultant solution was shaken in a dark room at room temperature for 30 min, and the absorption at a wavelength of 517 nm was read using a spectrophotometer (MODEl: UV2100 PC). In order to prepare the control, the above method was applied, and only instead of the extract, 25 μl of 80% methanol were used (Brand‐Williams et al., 1995). The percentage of antioxidant capacity was calculated through the following equation:

$$\text{DPPHsc \textbackslash{}\%}\;=\frac{{\left(\text{Abs control}\right)}_{t=Xmin}‐{\left(\text{Abs sample}\right)}_{t=Xmin}}{{\left(\text{Abs control}\right)}_{t=Xmin}}\times 100$$

Abs control, control absorption rate; Abs sample, sample absorption rate.

### 2.7 The measurement of antioxidant capacity through FRAP

Methanol extract and 3 ml of fresh FRAP (300 mM sodium acetate buffer with 3.6 acidity, TPTZ) were mixed together. The resultant mixture was placed in a hot water bath (37℃) for 30 min, and its absorbance was read at a wavelength of 593 nm using a spectrophotometer in comparison with the control. Iron sulfate was applied to draw the standard curve, and the results were presented in µmol Fe^++^/g dw (Žugić et al., 2014).

### 2.8 The measurement of total carotenoids and chlorophyll a and b

To measure carotenoids and chlorophyll, 0.05 g of dry tissue with 5 ml of methanol was chilled in a Chinese porridge and homogenized in an ice bath. Then, 1 g of sodium sulfate without water was added to the resultant homogenate and clarified using filter paper. The methanol‐filtered solution was infused to a volume of 10 ml and centrifuged at 6000 g for 10 min. The upper phase of separation and absorbance of the solution was measured at 666, 653, and 470 nm wavelengths in comparison with the control. Methanol was used as a control. The content of carotenoids and chlorophyll a and b and total chlorophyll for each extract were calculated using the following formulas (Lichtenthaler, 1987):

Chl_a_ = 15.65 A_666_–7.340 A_645_.

Chl_b_ = 27.05 A_653_–11.21 A_666_.

TCC = 1000 A_470_–2.270 Chla–81.4 Chl_b_ 0.227.

### 2.9 Cannabinoids extraction and GC‐Ms analysis

2 g of the plant was soaked in 10 ml of n‐hexane, and then it was sonicated. The determination of cannabinoids was performed by Ph‐5 fused silica column (10 m × 0.1 mm i.d; 0.40 μm film thickness) coupled with Thermo‐UFM ultra‐fast gas chromatograph. Conditions: injection temperature: 285°C, carrier gas helium (32 cm/s a linear velocity), detector (FID) temperature: 285°C, and temperature program: 60°C for 3, 60–280°C (80°C/min). The extracts were manually injected into the GC without dilution. The concentration of metabolites was investigated using the area normalization method. Different Gas chromatography/mass spectrometry (Varian 3400 GC‐MS, Markham, Ontario, Canada) coupled with a silica column (30 m × 0.25 mm i.d; 0.25 μm film thickness) were tested, in order to obtain the final optimized test. The oven temperature was programmed at 100°C for 5 min, increased by 10°C/min to 130°C, increased by 7°C/min to 270°C, and held to 270°C for 3 min. Helium was used as the carrier gas at a flow rate of 1.5 ml/min. 0.1% solution of extract was prepared in hexane, and 1 μL of this solution was injected. The injector was operated in split mode (20:1 split ratio) at a temperature of 270°C. Conditions: oven temperature was programmed at 50–240°C (4°C/min), transfer line 260°C. Helium carrier gas was used at a flow rate of 31.5 cm/s in split ratio 1:60, and electron impact (EI) ionization at 70 eV, scan time 1 s, and mass range of 40–300 amu. The 2 μl oils were diluted in 2 ml dichloromethane; then, 2 μl of each sample was manually injected to GC/MS. Identification of compounds was attained by comparing the mass spectra of the recorded chromatographic peaks with the SATURN data system database (Sparkman, 2005).

### 2.10 Statistical analysis

Data were analyzed using SAS 9.13 software in a completely randomized design with four treatments and three replications. Comparison of mean data was also done by Duncan's method.

## 3 RESULT AND DISCUSSION

### 3.1 Total phenolic content (TPC) and total flavonoid content (TFC)

**FIGURE 1:**
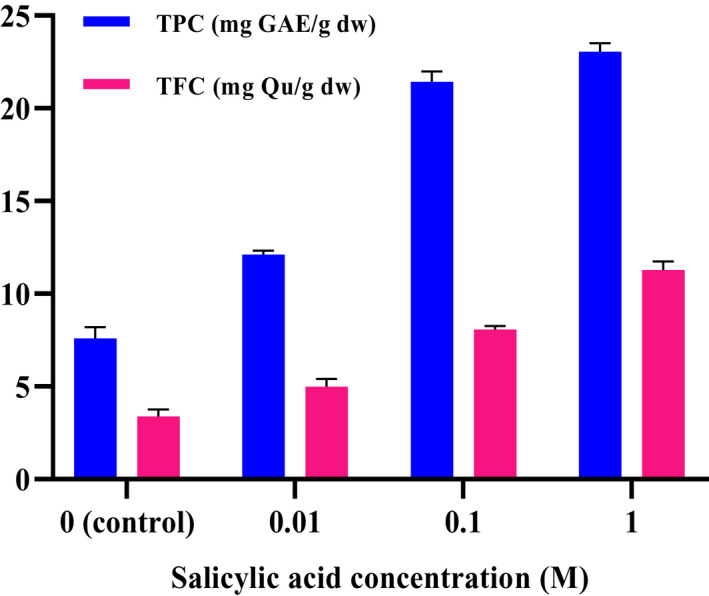
Effect of different concentrations of salicylic acid on total phenol content (TPC) and total flavonoid content (TFC) of *C. sativa*

The results demonstrated that total phenolic and total flavonoid contents in *C. sativa* have been affected by various concentrations of salicylic acid (*p* < .01). By applying different SA concentrations as foliar treatment, the TPC and TFC values in *C. sativa* plant increased from 7.59 to 23.06 mg GAE g^−1^ DW and from 3.38 to 11.28 mg Que g^−1^ DW, which are presented in Figure 1. The highest TFC and TPC were obtained in 1 M treatment, whereas the lowest TFC and TPC were obtained in control plants.

Sánchez‐Chávez et al. (2011) reported an increase in total phenolic and flavonoid contents in *Capsicum annuum* fruits with exogenous salicylic acid spray applications, whereas Vázquez‐Díaz et al. (2016) demonstrated a greater concentration of phenolic compounds in *Solanum lycopersicom* due to the increase in salicylic acid doses. The results of the present study are in agreement with the latter. Phenylalanine ammonium lyase (PAL), as the first committed enzyme in the phenylpropanoid metabolism, plays a key role in the biosynthesis of phenolic compounds in herbs (Ejtahed et al., 2015). Saba et al. (2012) indicated a significant positive correlation between content of polyphenols and activity of PAL enzyme. Moreover, it was found that salicylic acid stimulates the accumulation of hydrogen peroxide (H_2_O_2_), which induces a higher activity of PAL enzyme, responsible for the biosynthesis of polyphenols (Hao et al., 2014). The result of present study revealed that salicylic acid application achieved a positive effect on phenolic compounds in *Cannabis*. Probably, exogenous application of salicylic acid regulates the production of polyphenols via stimulating PAL enzyme activity. Moreover, *Cannabis* sprayed with salicylic acid indicated significantly greater amounts of total flavonoids than that in control plants. Chalcone synthase is considered to be the key enzyme for flavonoid biosynthesis of plants. According to previous studies on other herbs, the exogenous application of SA led to enhanced chalcone synthase activity (Gazdik et al., 2008).

Furthermore, application of SA regulates the jasmonate signaling pathway, which in turn mediates the elicitor‐induced accumulation of flavonoid compounds (Khalili et al., 2009). Sun et al. (2016) reported that application of salicylic acid on *Artemisia vulgaris* causes alterations in values of antioxidants, phenolics and in the activity level of genes associated with flavonoid synthesis.

### 3.2 Antioxidant capacity (FRAP and DPPH assays)

**FIGURE 2:**
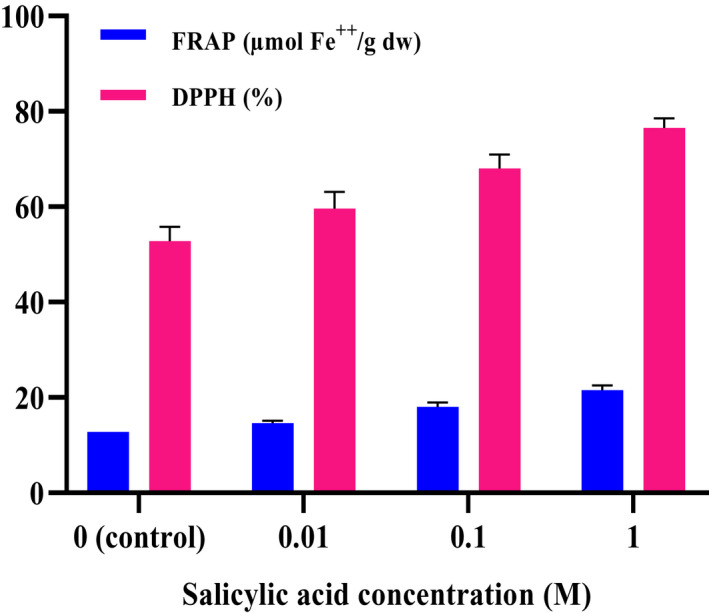
Effect of different concentrations of salicylic acid on antioxidant capacity of *C. sativa* by FRAP and DPPH assay

The results indicated that antioxidant capacity by DPPH and FRAP assays in *C. sativa* was affected by various concentrations of salicylic acid (*p* < .01). By applying different SA concentrations as foliar treatment, the antioxidant capacity in *C. sativa* plant increased from 12.80 to 21.58 µmol Fe^++^g^‐1^ DW (FRAP) and from 52.80% to 76.58% (DPPH), which are exhibited in Figure 2. The highest antioxidant capacity was obtained in 1 M treatment, whereas the lowest antioxidant capacity was found in control plants.

SA is involved in various physiological processes in plants through different signaling pathways. It increases the capacity of the antioxidant system by inducing anti‐oxidative enzymes activity or changing expression of some genes (Janda & Ruelland, 2015; Kang et al., 2013). In this study, antioxidant capacity of *Cannabis* aerial parts was enhanced as a consequence of salicylic acid treatment. Previous researches’ findings that demonstrated the positive role of exogenous salicylic acid treatment on raising antioxidant capacity are supporting our results (Osama et al., 2019). This increment can be justified with the enhancement of TPC and TFC. Zainol et al. (2003) reported that total phenolic content had remarkable antioxidant capacity. Other researchers also stated that with increasing polyphenols, antioxidant activity increased significantly (Rosicka‐Kaczmarek, 2004; Zhao et al., 2014). Results of current study suggest that the TFC and TPC increased due to the application of SA might be responsible for a large proportion of antioxidant capacity by FRAP and DPPH assays. The exogenous salicylic acid application increased antioxidant defense responses by inducing antioxidant enzymes in some plant species such as *Tanacetum parthenium* (Mallahi et al., 2018), *Lycopersicon esculentum* (Hayat et al., 2008), and *Dianthus superbus* (Ma et al., 2017). It was shown that the effect of salicylic acid application on increment antioxidant activities was related to its concentration in sprayed plants.

### 3.3 Photosynthetic pigments (TCC, Chl a, Chl b)

**FIGURE 3:**
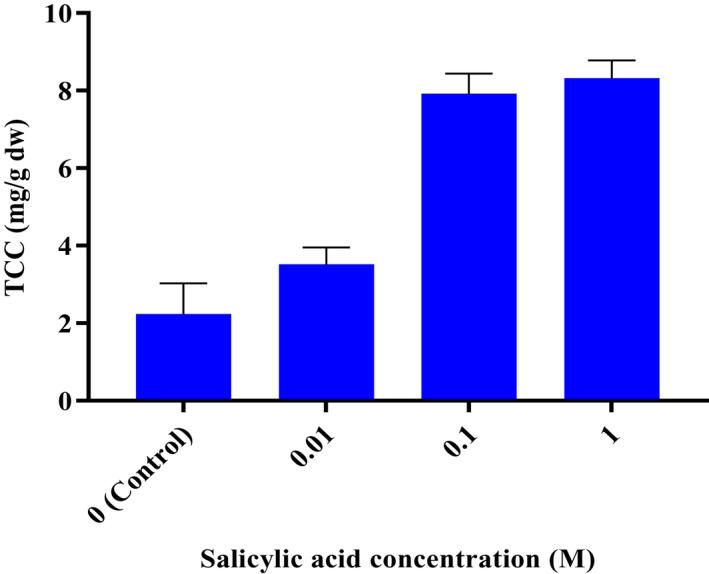
Effect of different concentrations of salicylic acid on total carotenoid content (TCC) of *C. sativa*

**FIGURE 4:**
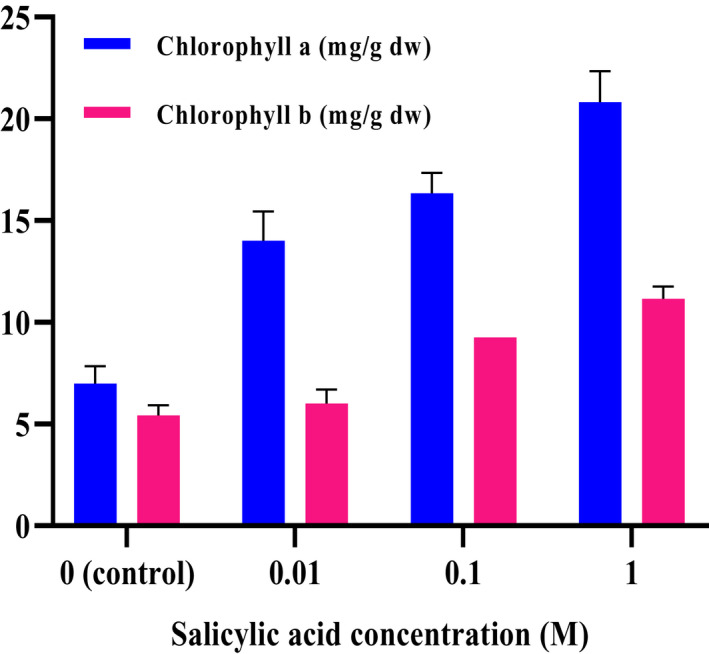
Effect of different concentrations of salicylic acid on chlorophyll a and b of *C. sativa*

The results indicated that TCC, Chl a, and Chl b in *C. sativa* were influenced by various concentrations of salicylic acid (*p* < .01). By applying different concentrations salicylic acid as foliar treatment, the TCC, Chl a, and Chl b in *C. sativa* plant increased from 2.24 to 8.32 mg/g DW, from 6.99 to 20.83 mg/g DW, and from 5.43 to 11.15 mg/g, which are presented in Figure 3 and Figure 4, respectively. The highest TCC, Chl a, and Chl b were obtained in 1 M treatment, whereas the lowest TCC, Chl a, and Chl b were found in control plants.

Several recent researches indicated that salicylic acid is a strong regulator of chlorophyll content and photosynthesis system in plants by influencing carotenoid and chlorophyll composition (Fariduddin et al., 2003; Gao et al., 2012). The salicylic acid application induces an increase in performance of the photosynthetic apparatus in plastids, resulting in high level of chlorophylls in leaf tissues (Dong et al., 2011). In soybean plants, spraying of salicylic acid increases the pigment composition, as well as the photosynthetic efficiency (Fahad et al., 2014). Spraying *Cannabis* with salicylic acid caused excitation of the total photosynthetic pigments TCC, Chl a, and Chl b in leaves compared with control samples (Figures 3 and 4). These findings are in agreement with Jaiswal et al. (2014), Idrees et al. (2010), Moharekar et al. (2003), and Jakhar and Sheokand (2015). The SA protects cell membranes against lipid peroxidation by scavenging free radicals (Antonic et al., 2016). It was shown that salicylic acid application can effectively alleviate pigment damage in various plant species (Borsani et al., 2001; Li et al., 2014; Ma et al., 2017).

### 3.4 Cannabinoid compounds

The amounts of individual cannabinoid and other compounds in the various treatments of SA are shown in Table 1. The results indicated that cannabinoid compounds in *Cannabis* were significantly influenced by the different concentrations of SA (*p* < .01). The major cannabinoid in the analyzed extracts was CBD (19.91%–37.81%), followed by Δ^9^‐THC (10.04%–22.84%) and CBL (nd‐14.78%). The highest CBD (37.81%) and Δ^9^‐THC (22.84%) were obtained in 1 M of SA, whereas the lowest CBD and Δ^9^‐THC were found in control and 0.01 M of SA, respectively. The CBL component (14.78%) was detected only in control and not in other treatments.

**TABLE 1:** Effect of different concentrations of salicylic acid on individual cannabinoids and other compounds in *C. sativa*

Code	Compound	Salicylic acid concentration (M)			
		0 (Control)	0.01	0.1	1
1	Octane	0.35	7.76	1.76	0.77
2	Caryophyllene	2	6.63	5.8	2.24
3	β‐Caryophyllene	0.5	1.79	1.46	0.67
4	Naphthalene	–	1.34	–	–
5	5‐Azulenemethano	0.35	–	–	–
6	Bisabolol	–	–	–	0.38
7	Phytol	0.84	–	–	0.35
8	Dibutyl phthalate	0.31	–	–	–
9	Docosane	1.02	1.75	7.18	1.88
10	Pentacosane	2.52	4.55	–	–
11	Hexatriacontane	4.53	5.42	–	–
**12**	**Cannabidiol (CBD)**	**19.91**	**20.46**	**24.61**	**37.81**
**13**	**Cannabicyclol (CBL)**	**14.78**	–	–	–
14	Heptacosane	6.68	6.42	2.4	6.96
15	Octacosane	–	–	–	13.41
**16**	**Δ0.9‐Tetrahydrocannabinol (Δ^9^‐THC)**	**10.04**	**6.5**	**23.79**	**22.84**
17	Cannabigerol	–	–	–	0.58
18	Nonacosane	–	–	1.08	0.34
19	Squalene	2.91	2.7	3.71	2.26
20	Hentriacontane	6.46	5.14	6.54	2.22
21	Dotriacontane	0.98	7.16	–	–
22	Pentatriacontane	21.23	14.38	15.82	0.36
23	Vitamin E		5.06	–	–
24	Tetratetracontane	3.74	2.93	2.57	0.36
25	(‐)‐standishinal	–	–	3.27	–

Note

Major cannabinoid compounds of *Cannabis sativa* are highlighted in bold.

Nowadays, major cannabinoids (THC and CBD) have become considerably more potent for therapeutic aims. The type and concentration of elicitors can be a stimulant for the production of phytochemicals in *C. sativa* species, which shows the importance of CBD and THC in the pharma industry. The results showed that different concentrations of SA lead to changes in the content of THC and CBD metabolites. Elicitor concentration plays a key role in inducing the expression of major genes and subsequent production of phytochemicals (Flores‐Sanchez et al., 2009). The findings of the current study revealed that foliar sprays of SA have positive effects on the improvement of the cannabinoid compounds. In recent years, SA has been recognized for its important role as an activating signal for a variety of plant defense responses. It also stimulates specific biosynthetic pathways of natural compounds in plants (Bulgakov et al., 2002; Sirvent & Gibson, 2002). In addition, SA, as a cheap, safe, and commonly available biological molecule, appears to be a viable option for modifying/ synthesizing valuable secondary metabolites in *Cannabis*. Several genes and enzymes (CBDAS, THCAS, OLS, and PT) play a remarkable role in the synthesis and accumulation of cannabinoids (CBD and THC). The expression of these genes varies in various SA concentrations (Jalali et al., 2019). According to the results of Jalali et al. (2019), responses of various gene expression were obtained after using with salicylic acid. Exogenous treatment of SA resulted in increment in THCAS expression. Elicitor concentration plays an important role in stimulation of major genes and consequently a significant increase in the production of phytochemicals (Giri & Zaheer, 2016). There were similar studies in other plants which reported SA has a dose‐dependent positive effect on cannabinoid compounds, which was consistent with the results of the present study.

## 4 CONCLUSION

The objective of the current research was to apply an effective elicitor to improve antioxidant capacity, phytochemicals, and cannabinoid compounds of *C. sativa*. In general, results of the current study revealed that the *C. sativa* sprayed with SA had great antioxidant potential, phytochemical contents (TFC, TPC, TCC, and chlorophylls), and cannabinoid compounds. The elicitor SA (especially 1 M) was able to improve antioxidant capacity, phytochemical and cannabinoid compounds.

## CONFLICT OF INTEREST

None.

## AUTHOR CONTRIBUTION

**Elham Mirzamohammad:** Writing‐original draft (equal). **Abolfazl Alirezalu:** Methodology (equal); Project administration (equal); Supervision (equal). **Kazem Alirezalu:** Writing‐review & editing (equal). **Asadaolah Norozi:** Data curation (equal); Formal analysis (equal). **Afsaneh Ansari:** Data curation (equal); Formal analysis (equal); Investigation (equal).

## Data Availability

The data that support the findings of this study are available from the corresponding author upon reasonable request.
